# Feasibility of a paediatric radiology escape room for undergraduate education

**DOI:** 10.1186/s13244-020-00856-9

**Published:** 2020-03-19

**Authors:** Chantal Liu, Raeesa Patel, Bukola Ogunjinmi, Corey Briffa, Miranda Allain-Chapman, Josephine Coffey, Neha Kallam, Marco Shiu Tsun Leung, Annabelle Lim, Sabina Shamsad, Farah El-Sharnouby, Emily Tsang, Jennifer Whitehead, Josephine Bretherton, Lauren Ramsay, Susan C. Shelmerdine

**Affiliations:** 1 St. Georges, University of London, Cranmer Terrace, London, SW17 0T UK; 2 University College London Medical School, 74 Huntley Street, London, WC1E 6BT UK; 3 Great Ormond Street Hospital for Children NHS Foundation Trust, London, WC1N 3JH UK; 4 UCL Great Ormond Street Institute of Child Health, WC1N 1EH, London, UK

**Keywords:** Radiology, Paediatric, Teaching, Escape room, Gamification, Education

## Abstract

**Objectives:**

To develop a paediatric radiology themed escape room session for undergraduate education and secondly, to determine participant satisfaction and improvement in knowledge.

**Methods:**

A paediatric radiology escape room with accompanying tutorial was developed around key learning objectives set within the RCR and ESR undergraduate curriculum. Students were recruited from two different universities and undertook the escape room themed teaching. An 8-question single best answer (SBA) test was completed before, immediately after and at 2 weeks post-teaching to determine participant improvement and retention of knowledge. The general feedback was also collected.

**Results:**

The escape room sessions were held three times, for 19 students (6–7 students per session). All groups completed the escape room in ≤ 20 min. Students enjoyed the experience, assigning an average satisfaction score of 9.4/10 (range 7–10). The majority (17/19, 89.5%) preferred this method of teaching to a lecture-based tutorial alone, although all said they found the tutorial component useful. For the SBA test, there was an average increase in 3.6 marks (range 1–6 marks) per participant between before and after the escape room. This improved knowledge was mostly sustained after 2 weeks, with an average increase of 3.4 marks difference (range 1 to 6) per participant compared to before the teaching.

**Conclusions:**

A paediatric radiology themed escape room is a feasible teaching method, enjoyed by participants and associated with an increase in radiological knowledge. Further work with larger sample size and direct comparison with other traditional teaching methods is required.

## Keypoints

- A paediatric radiology themed escape room is a feasible teaching method.
- Students enjoyed the escape room, and most preferred it over didactic lectures.
- Improvement in paediatric radiology knowledge was maintained after the teaching.

## Introduction

The use of interactive teaching and games in healthcare education has a positive impact on the learning process [1], with most junior doctors and undergraduate students stating a preference for a small group, interactive teaching for radiological education [2, 3]. Recently, the ‘escape room’ concept has emerged as a novel method for delivering interactive teaching. An ‘escape room’ typically comprises of several physical games in a metaphorically ‘locked’ classroom. Participants are encouraged to communicate and work collaboratively to solve the puzzles, which will eventually enable them to ‘unlock’ the room and escape [4, 5]. A time limitation is commonly present to introduce an element of stress, excitement and competition. This teaching method has been shown to be feasible and enjoyable when developed and tested on radiology trainees [6], as well as undergraduate students on a variety of healthcare topics (e.g. nursing [7, 8], pharmacy [9], surgery [10, 11] and dermatology [12]).

Given the variability of undergraduate radiology education delivered in universities across the UK [13] and Europe [14], the fact that most junior doctors do not feel confident interpreting paediatric radiographs [3] with added workforce issues relating to both paediatric radiology [15], paediatric emergency services [16] and paediatric medicine [17], it is likely that having some exposure to paediatric radiology at an undergraduate level may prove beneficial in later medical practice [18]. As puzzles within an escape room are problem-based and require communication and team-working skills—considered intrinsic parts of the way in which adults learn [19], we hypothesised that this teaching method would lend itself well to an undergraduate paediatric radiology tutorial.

The aim of this study was therefore two-fold: firstly, to develop a paediatric radiology themed escape room session for undergraduate education and secondly, to assess participant satisfaction and improvement in paediatric radiological knowledge.

## Materials and methods

Ethical approval was not required for this study, assessing the anonymised student feedback and knowledge from a teaching event. All students provided consent for the use of their anonymised feedback in this study. No external funding was provided for this project.

### Escape room design

A paediatric radiology themed escape room session and 8-question single best answer (SBA) test was devised by the senior author (S.S.), a paediatric radiologist (10 years of radiology experience, 5 in paediatric radiology). Both the SBA test and escape room were constructed with specific learning objectives in mind, mapped to outcomes from the Royal College of Radiologists (RCR) [20] and European Society for Radiologists (ESR) [21] undergraduate radiology curriculums. These objectives included delivering knowledge on (1) radiation protection, (2) fracture detection on paediatric radiography and (3) emergency findings on paediatric chest radiographs (e.g. consolidation, pneumomediastinum, pneumothorax) (see Additional file 1: Figure A1). A copy of the SBA test, with answers in the caption, is also provided in Additional file 1: Figure A2.

**Fig. 1:**
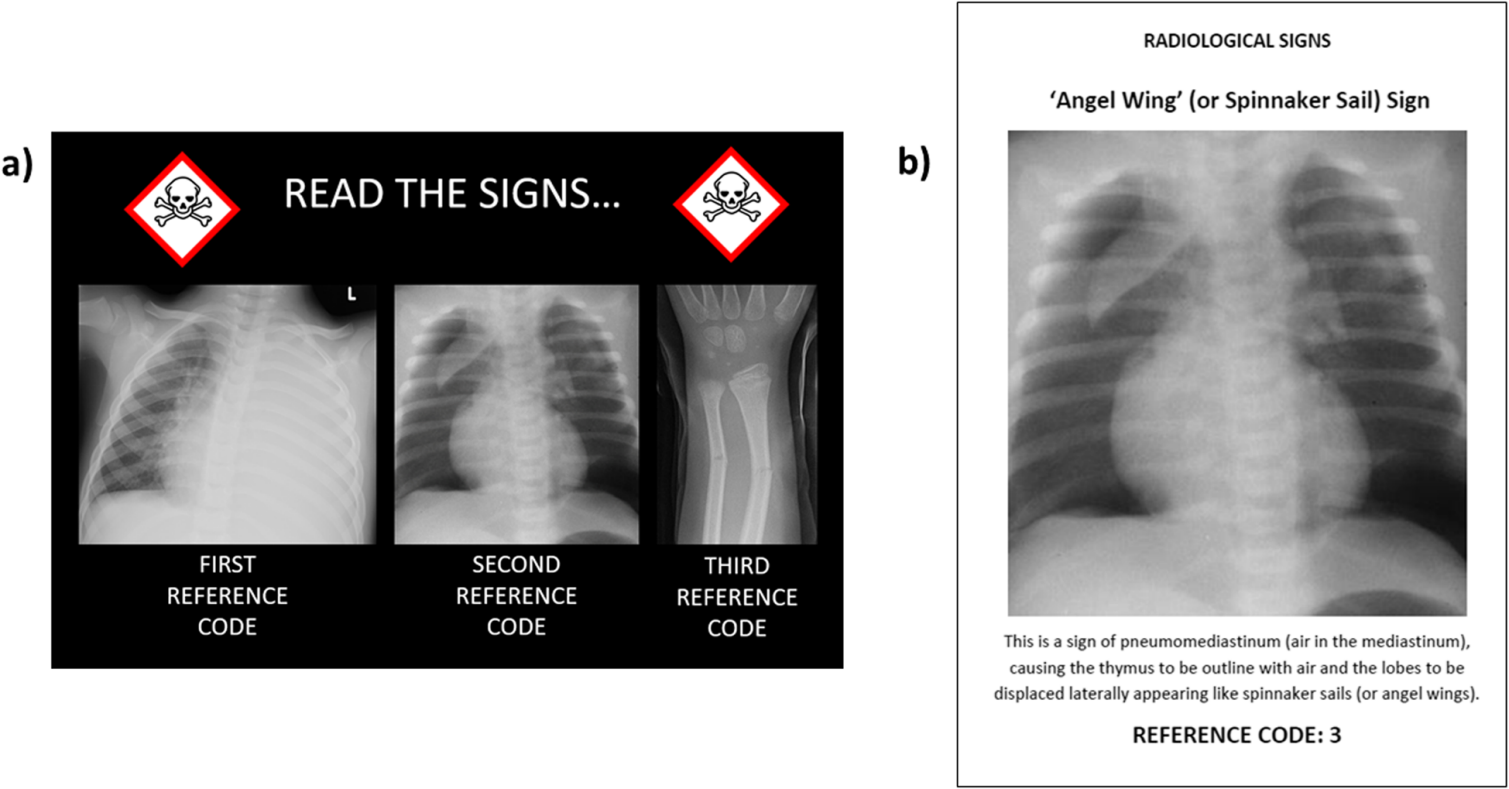
‘Read the signs’: an example of one of the puzzles in the escape room. **a** A poster detailing three different paediatric radiographs with abnormalities is placed on one of the walls of the room. The students need to find a hidden folder of ‘radiology signs’ somewhere in the room containing 20 different imaging signs and match the images on the poster to that in the folder to solve the three number combination code. **b** An example of one of the pages in the clear plastic presentation folder matching the second image on the poster. In this example, the second number for the combination code would be ‘3’

**Fig. 2:**
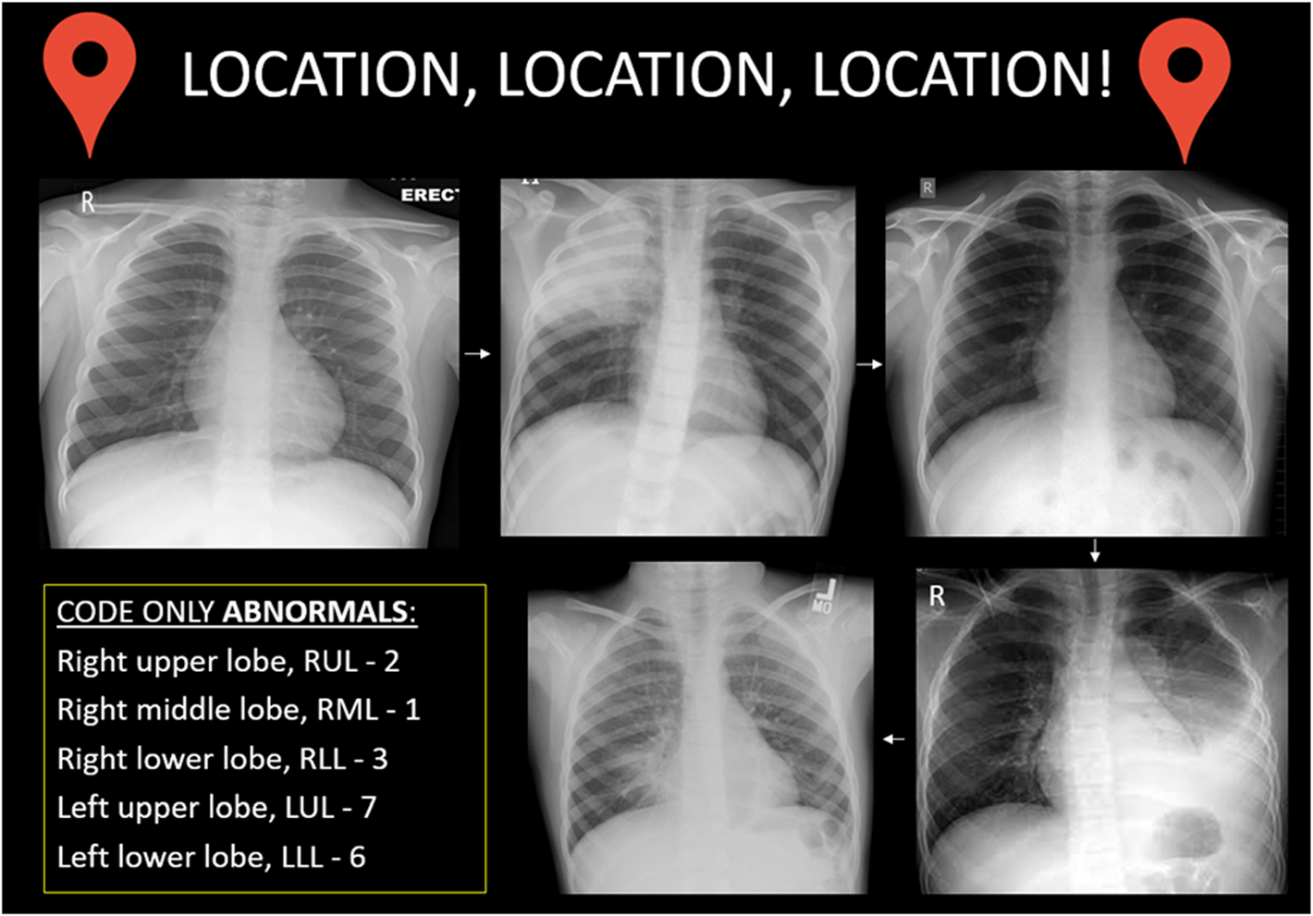
‘Location, location, location’: another example of a puzzle in the escape room. The participants need to identify whether a chest radiograph is normal or abnormal. If abnormal, then the location of the consolidation should be determined and matched to the number code given in the box in the bottom left corner of the poster. For example, in this game, the three number combination is 2, 6 and 1

**Fig. 3:**
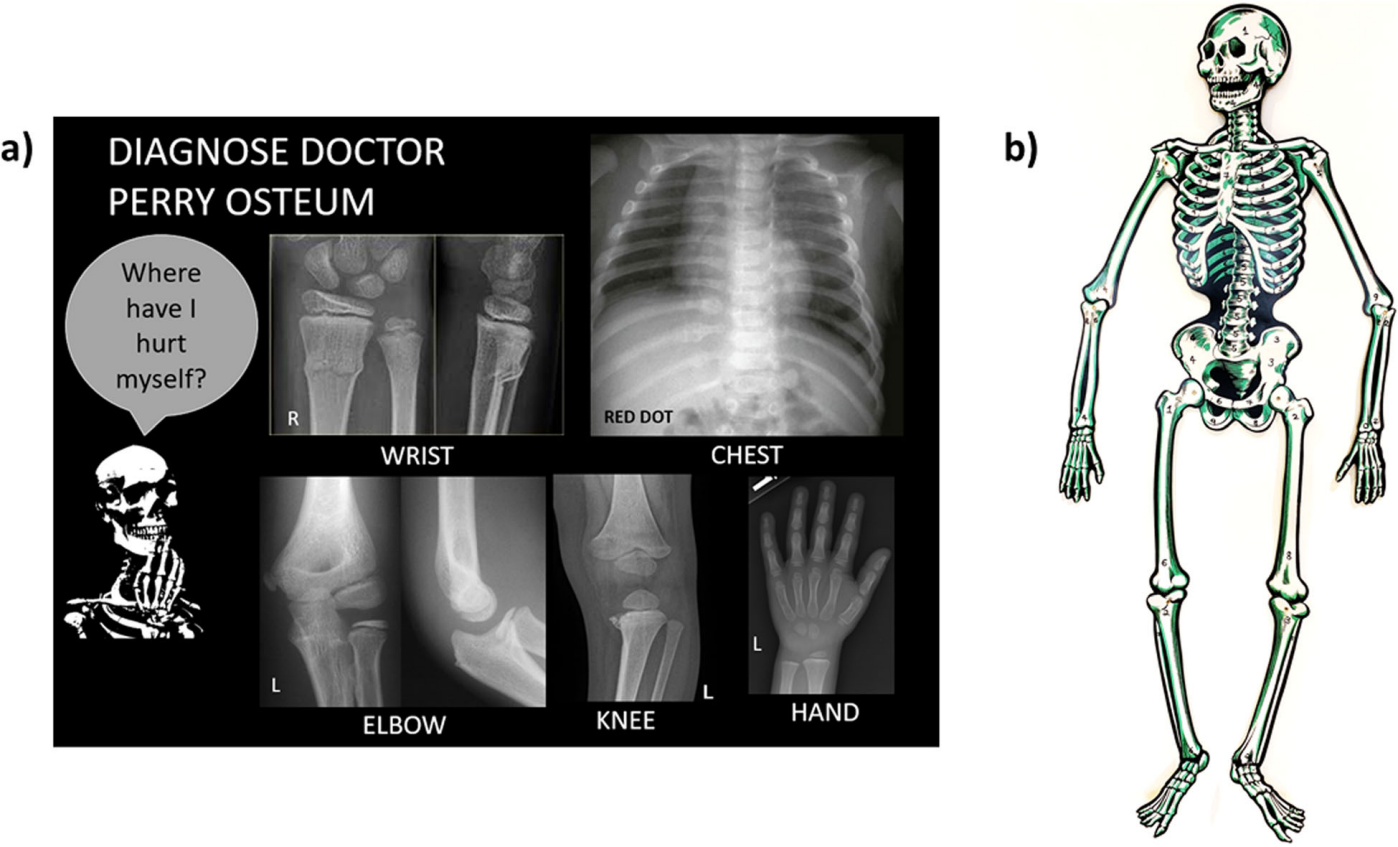
‘Diagnose Doctor Perry Osteum’: another example of a puzzle in the escape room. **a** A poster of various body parts some with and some without fractures are shown. The participants need to identify which bones have an abnormality and match it to the (**b**) large paper skeleton in the other corner of the room. This skeleton has numbers written on every bone, and only by correlating the correct bone and laterality can the students resolve the three-number combination. In this example, the bones to be interrogated on the skeleton would be the distal right radius, posterior right ribs and proximal left tibia

**Fig. 4:**
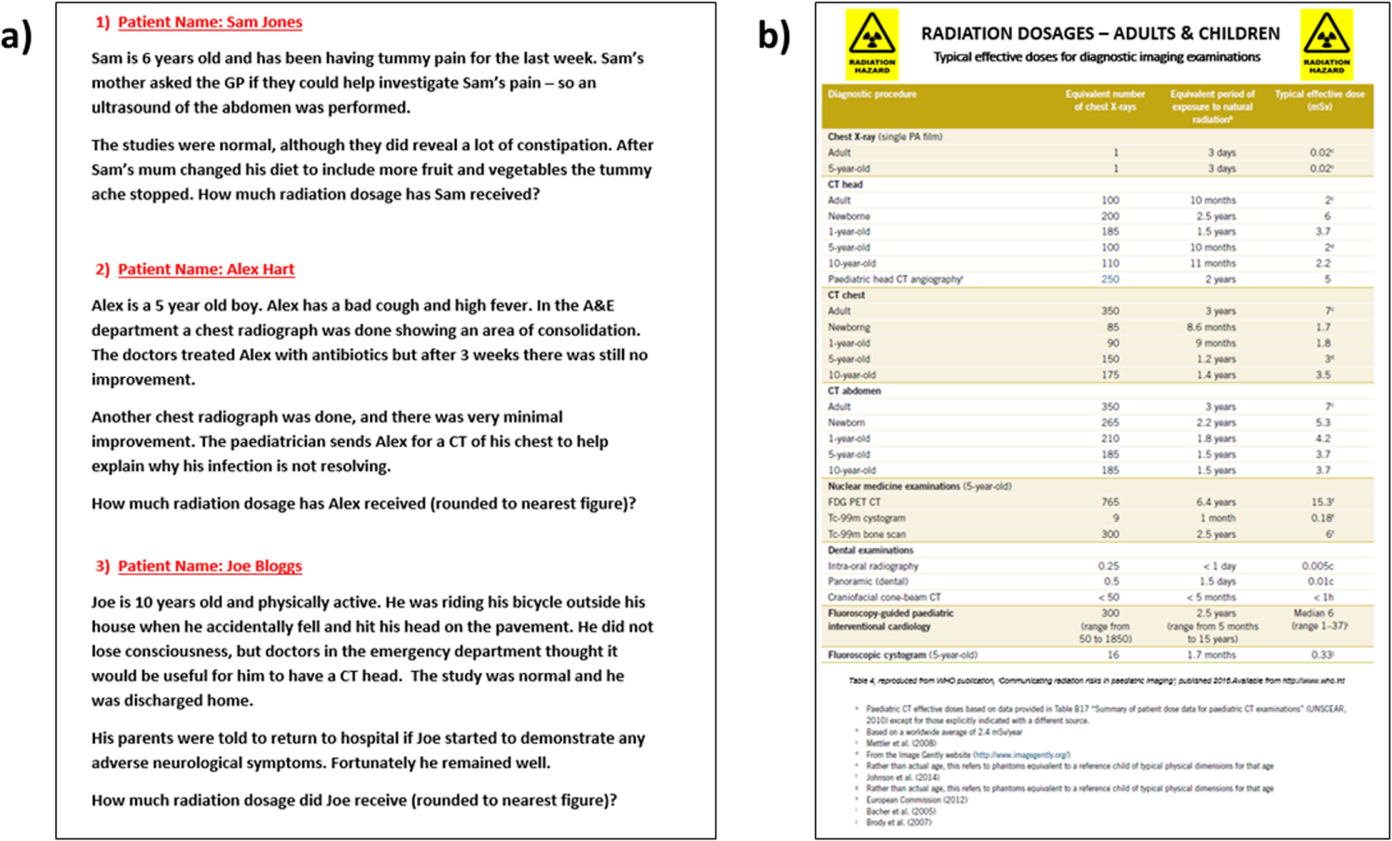
‘Radiation dosages’ puzzle: In this puzzle (**a**) the patient scenarios are placed on the wall, with a list of (**b**) radiation dosages on another wall, based on the WHO 2016 publication, ‘Communicating radiation risks in paediatric imaging’ [27]. Students are required to calculate the radiation dosages from the different radiology modalities and examinations to solve the three-number combination. In this example, the combination code was 0, 3 and 2

**Fig. 5:**
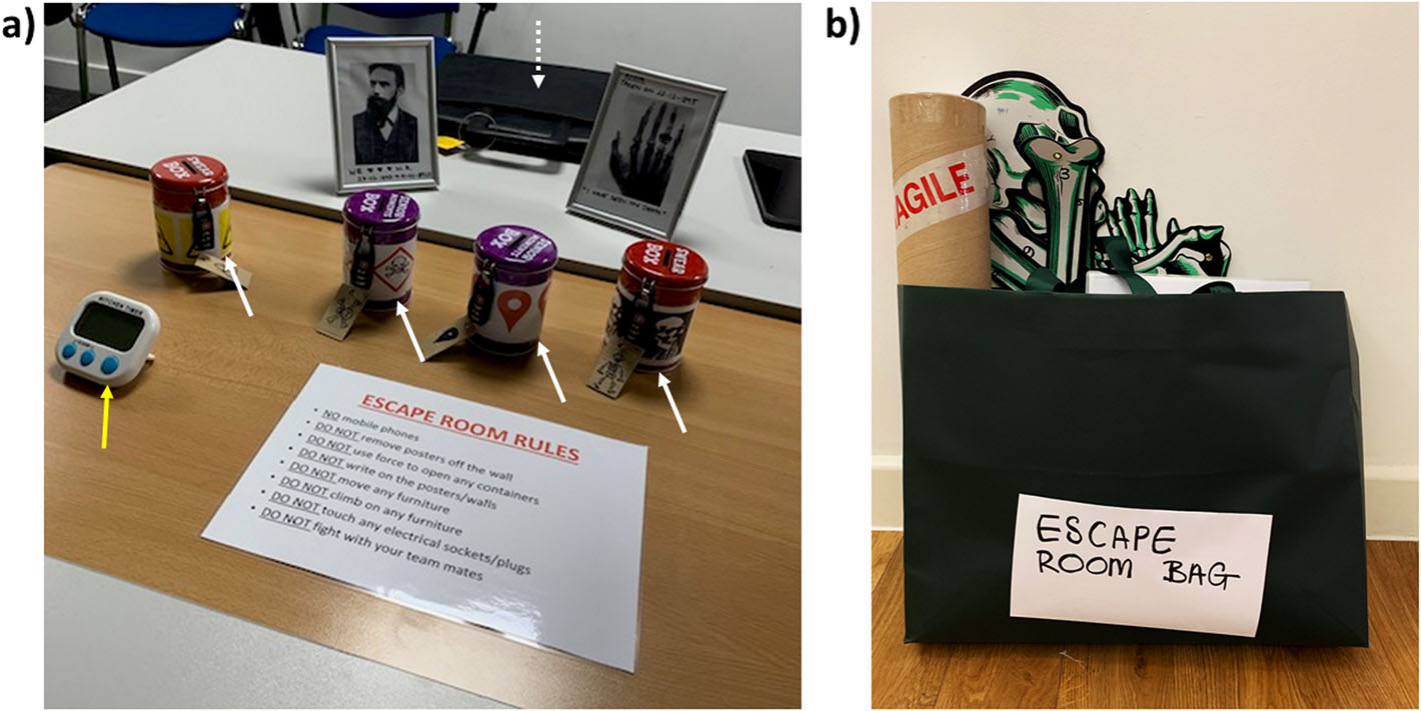
Equipment in the escape room. **a** The equipment on the central desk included a copy of the escape room rules, the kitchen timer to time the participants (yellow arrow), the four tin containers (white arrow) which were each locked with a three-number combination lock and contained a note for the final crossword puzzle, and clues on how to unlock the briefcase containing the puzzle (dashed white arrow). Photo frames containing images of Roentgen and an early example of a hand radiograph were not part of the escape room puzzles, and only placed on the table for decoration. **b** All the equipment was easily transportable between teaching sites within a single carry bag

**Fig. 6:**
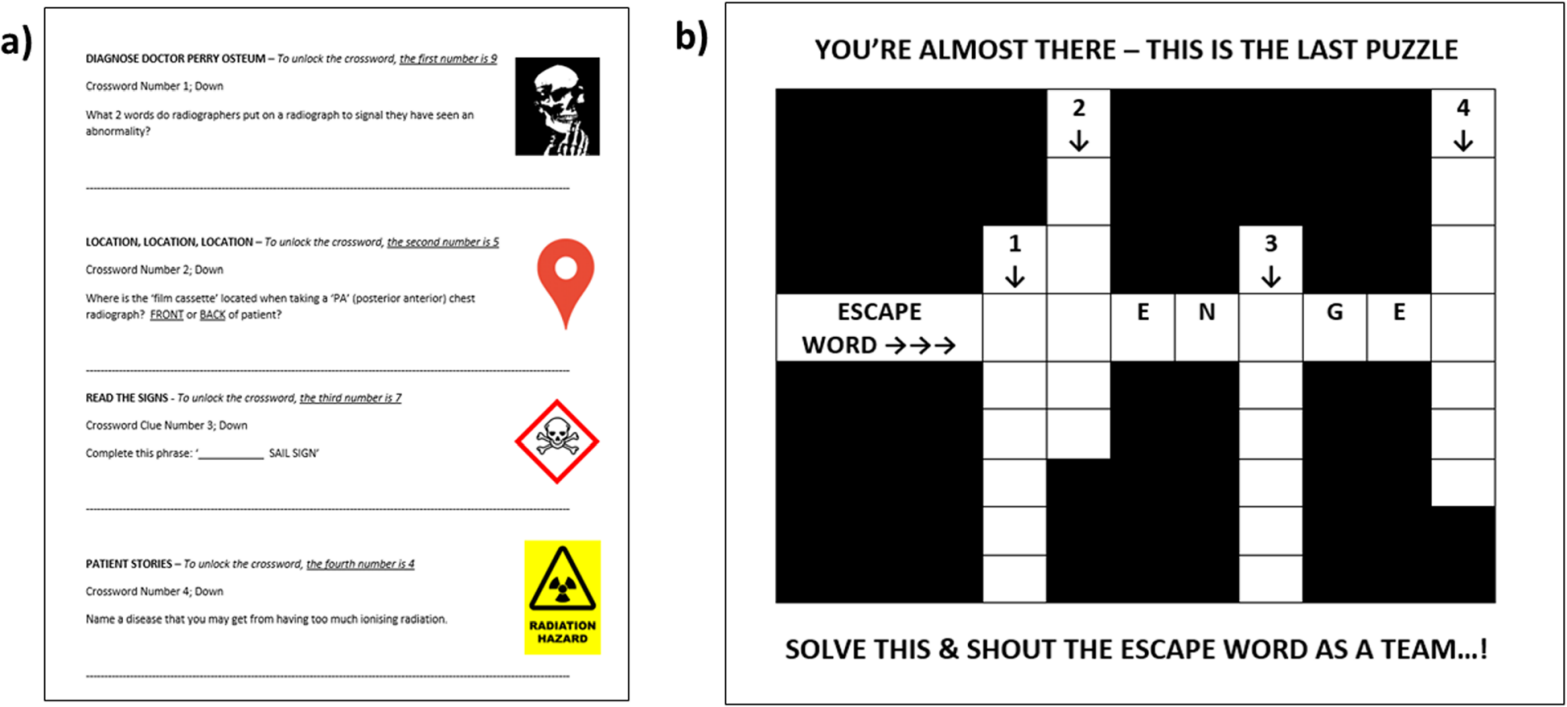
Crossword puzzle. **a** This image demonstrates the four different notes that were contained within the four containers shown in Fig. 5. They provide clues to the crossword puzzle (**b**). The answers to the crossword are 1—RED DOT; 2—FRONT; 3—THYMIC; 4—CANCER. The escape room word was ‘ROENTGEN’

**Fig. 7:**
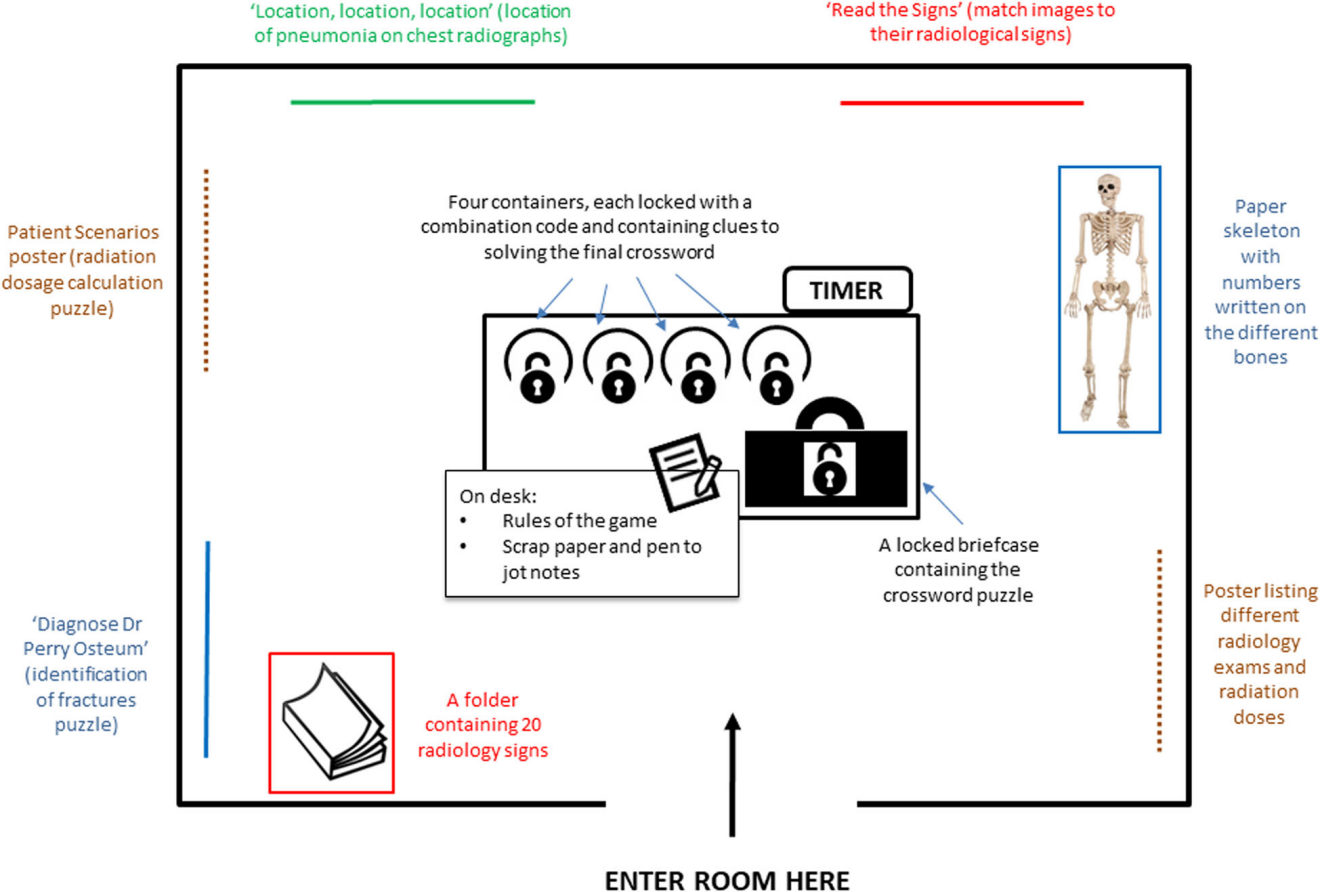
The layout of the escape room. The puzzles listed in Figs. 1, 2, 3 and 4 are colour coded in this image. Those with more than one component are intentionally placed on opposite sides of the room to force students to interact with each other and communicate findings. The escape room rules read to students prior to the activity had already informed them that removing posters from the wall or use of mobile phones was not allowed

The escape room consisted of four radiological ‘puzzles’ (relating to the objectives above), that could be solved in any order (Figs. 1, 2, 3 and 4). After solving each of these puzzles, a three-number combination code would be revealed allowing participants to unlock one of four tin containers in the room (Fig. 5). Within each container was a note, upon which a single number and crossword clue were written. Only by unlocking all four containers (i.e. by solving all four puzzles) could the combination of numbers on the notes provide a code to unlock a briefcase containing a crossword puzzle. In order to ‘unlock’ the room, participants would need to solve the crossword to reveal a secret word, which they shout in unison in order to escape (Fig. 6). A layout of the room is shown in Fig. 7.

Prior to running the undergraduate teaching session, a ‘practice run’ was piloted on two paediatric radiology fellows active in undergraduate teaching (J.B., L.R.—each with 6 years of radiology experience and 1 year paediatric radiology) to identify errors in the design, feedback on level of difficulty and to inform estimated time required to complete the puzzles. Both radiology fellows were blinded to the escape room design prior to the practice run.

### Undergraduate student recruitment

Undergraduate medical students from two different universities (University College London, UCL and St George’s University of London, SGUL) were recruited. The escape room teaching sessions were held on two separate dates, both in October 2019.

A single escape room based teaching session was held for the UCL students, which took place during working hours as part of their weekly compulsory undergraduate medical teaching programme at the senior author’s institution, organised by the in-house postgraduate medical education department (Great Ormond Street Hospital, London). Two sessions of escape room based teaching were held for SGUL students, both taking place in the early evening as part of an undergraduate radiology society event, attended by students on a voluntary basis. In this second setting, the students attending were recruited by event posters and email, Facebook and social media alerts.

### Outline of the teaching session

Each teaching session lasted 90 min. In the first 10 min of the sessions, students completed the SBA test to gauge their baseline radiological knowledge. The rules of the escape room were then explained by the senior author (Additional file 1: Figure A3), and a fictional ‘backstory’ was delivered to set the mood (Additional file 1: Figure A4).

The students were then taken to the escape room (separate to where the briefing took place). Once in the room, a timer was started to add a competitive element. A time limit of 30 min was set. The senior author (SS) was present in the room for all sessions to provide general supervision and observation, but the students were not allowed to ask for any hints. Following the completion of the escape room, a 45-min radiology tutorial was delivered by the senior author (S.S.), covering a ‘walk-through’ of the key findings in the escape room puzzles and the aforementioned learning objectives.

At the end of the teaching, students engaged in a team photo, completed a feedback form of their experience (Additional file 1: Figure A5) and the same SBA test again. This SBA test was later hosted online as a ‘Google Forms’ questionnaire and the hyperlink emailed to students after 2 weeks to assess retention of knowledge. Two weeks was chosen an arbitrary time point, long enough to avoid students relying on immediate recall yet recent enough to remember the teaching session having taken place.

The students did not receive any individualised feedback regarding their test scores at any point during the study. The students were not pre-warned that their knowledge would be re-checked after and in 2 weeks’ time from the escape room teaching session.

### Data analysis

Data from the student feedback forms and the SBA test scores were transferred into an Excel spreadsheet (Microsoft, USA) and descriptive analyses using the total, mean and range of feedback scores and test scores were performed.

## Results

### Escape room design

Devising the escape room took place over a period of 2 weeks. This included sourcing the individual equipment for the room, preparing the puzzles and testing the design. The total estimated cost for the materials was £93.83 (Additional file 1: Figure A6), and all items could be easily transported within one bag (Fig. 5).

The practice run of the escape room took 10 min to set-up and was completed in 14 min by the two radiology fellows. They found the puzzles easy to understand and solve, and felt the level of difficulty would be appropriate for undergraduate teaching.

### Student demographics

Nineteen participants (6 from UCL, 13 from SGUL) completed the escape room across the three escape room teaching sessions. There were 7/19 (36.8%) male participants. Most of the participants were in their final undergraduate year (i.e. year 6) of training (8/19 (42.1%)), with others from year 5 (1/19, 5.3%), year 4 (3/19, 15.8%), year 3 (2/19, 10.5%), year 2 (2/19, 10.5%), year 1 (2/19, 10.5%) and one recently qualified foundation year 1 doctor (1/19, 5.3%).

None of the participants had previously received paediatric radiology teaching, although most (16/19, 84.2%) had prior general radiology teaching. Only three students (3/19, 10.5%) reported no prior radiology teaching. They were in years 1 and 2 of undergraduate study. Of the 16 students who had previous radiology teaching, the most common method for teaching were small group tutorials (11/16, 68.8%), with 6/16 (37.5%) receiving large group lectures and 5/16 (31.3%) having completed placements in a radiology department. Approximately, a third of students (6/19, 31.6%) had previously completed an escape room before (non-healthcare education-related).

### Escape room feedback

The escape room teaching session was run three separate times for different teams of participants. A 6-person team participated in the first two sessions, and 7-person team in the third session. The escape room was solved by all three teams within the 30 min time-limit, with the following times and participant seniority levels:

- Session 1: 14 min 57 s; six final years (year 6) students
- Session 2: 20 min; 1 year 1, one year 2, one year 3, two year 4 and one year 5 student
- Session 3: 16 min 28 s; one year 1, one year 2, one year 3, one year 4, two year 6 students and one recently qualified junior doctor (foundation year 1).

Feedback regarding overall enjoyment and the running of the escape room is provided in Table 1. The level of difficulty reported by the students for each of the puzzles is outlined in Table 2. Most students enjoyed the experience (average score of 9.4 out of 10 for enjoyment) and felt they had enough time to complete the task. They also found the number of students in the sessions to be adequate. Most students (17/19, 89.5%) reported that they would prefer having an escape room component to a teaching session than a longer tutorial instead; however, all students found the tutorial useful. Although the level of difficulty for each puzzle varied with students’ prior radiology knowledge, the average scores suggested an appropriate level of difficulty. Free text feedback comments after the teaching are included in the Additional file 1: Figure A7.

**Table 1:** Participant feedback on escape room enjoyment, set-up and difficulty of puzzles

Escape room feedback question	Response	Average score (for numerical answers)
How enjoyable was the escape room on a scale of 1–10; where 10 = best?	10 (*n* = 14, 52.6%) 9 (*n* = 1, 5.2%) 8 (*n* = 2, 10.4%) 7 (*n* = 2, 10.4%)	9.4
Was enough time provided to complete the room?	Yes (*n* = 19, 100%)	–
Were the number of team players in your session appropriate?	Yes (*n* = 19, 100%)	–
Ideally what would be the best number of players for such an activity?	7 (*n* = 8, 42.1%) 6 (*n* = 8, 42.1%) 5 (*n* = 2, 10.4%) 4 (*n* = 1, 5.2%)	6.2
Did you find the tutorial after the escape room useful?	Yes (*n* = 19, 100%)	–
Would you have preferred a longer tutorial instead of an escape room with tutorial?	No (*n* = 17, 89.5%) Yes (*n* = 2, 10.5%)	–

**Table 2:** Participant feedback on difficulty level of the escape room puzzles, scored on a scale of 1 to 10, where 1 = too easy; 5 = about right level; 10 = too difficult

Escape room puzzles	Average difficulty (range)
Matching images to radiology signs	4.5 (1–6)
Identification of lobar consolidation	4.7 (1–8)
Calculation of radiation dosages	4.3 (1–7)
Identification of fractures on radiographs	5.4 (4–8)
Crossword puzzle	5.5 (4–8)

### SBA test scores

All students completed the pre-escape room and immediate post-escape room SBA tests at the time of the teaching. The online SBA test was completed by 13/19 (68.4%) students 2 weeks after the teaching session.

The mean test score (out of 8) prior to the teaching was 3.7 (range 2–6), improving to 7.3 (range 4–8) immediately after the teaching session, and 7.3 (range 5–8) 2 weeks later. When assessing the difference in scores on a ‘per participant’ level, this corresponded to an average increase of 3.6 marks (range 2–6) between pre- and post-teaching sessions; but a slight reduction of 0.5 marks (range − 2 to + 1) between scores at post-teaching and 2 weeks later. The average difference between the pre-teaching (i.e. baseline) and 2 weeks test was an increase in 3.4 marks (range 1–6) per participant. Only 5/13 (38.5%) students performed worse at 2 weeks compared to their immediate post-session test. All participants scored higher immediately after, and at 2 weeks after the teaching session compared with their baseline marks.

The number of correct answers on a per-question level amongst the students is outlined in Table 3. Prior to the teaching session, the least number of correct responses were provided for questions asking about the silhouette sign-on radiography (3/19, 15.8%), naming a stochastic effect of ionising radiation (1/19, 5.3%) and recognising the radiographic sign for pneumomediastinum (4/19, 21.1%). After the teaching session, there was an improvement in the number of correct responses for all questions, although the one regarding the silhouette sign was still the least correctly answered (14/19, 73.7% correct). At 2 weeks post-teaching, the questions with the least correct responses were regarding the definition of a greenstick fracture and explanation of the silhouette sign (both 11/13, 81.8%). The only question to show a reduction in the overall percentage of correct answers between the pre-teaching and at 2 weeks later was for the definition of a greenstick fracture (89.5% correct pre-teaching versus 85% at 2 weeks). This was mainly due to two students choosing the incorrect answer at the post-2 weeks test—one was able to select the correct response immediately after teaching, but wrong response both at pre- and 2 weeks post-teaching; and another who appeared to get the correct answer before and immediately after teaching, but presumably forgot or accidentally selected the incorrect answer at the 2-week test.

**Table 3:** Number and proportion of each question in the single best answer (SBA) test which were answered correctly at each time interval

Question	Pre-escape room (*n* = 19)	Post-escape room (*n* = 19)	2 weeks later (*n* = 13)
Q1. Definition of silhouette sign	3 (15.8%)	14 (73.7%)	11 (85.0%)
Q2. Appearances of right middle lobe consolidation	15 (78.9%)	19 (100%)	12 (92.0%)
Q3. Definition of greenstick fracture	17 (89.5%)	19 (100%)	11 (85.0%)
Q4. Naming of a stochastic effect of ionising radiation.	1 (5.3%)	17 (89.5%)	12 (92.0%)
Q5. Identifying which imaging modality does not use ionising radiation.	14 (73.7%)	17 (89.5%)	12 (92.0%)
Q6. Naming the bony injury having a high association with suspected physical abuse.	8 (42.1%)	18 (94.7%)	12 (92.0%)
Q7. Naming a part of a child’s bone on a diagram.	9 (47.4%)	19 (100%)	12 (92.0%)
Q8. Understanding the spinnaker sign.	4 (21.1%)	16 (84.2%)	13 (100%)

## Discussion

A paediatric radiology themed escape room is a feasible, enjoyable and educational method for delivering undergraduate radiology education. All students improved their knowledge of paediatric radiology immediately after the teaching session, with sustained improvement after a 2-week period compared to baseline.

When comparing prior studies relating to healthcare education-themed escape rooms [4 ,6, 7, 10, 12, 22, 23] (Table 4), only Eukel et al. [22] have objectively measured student knowledge prior to and immediately after the teaching session. They found that the average test score (based on a 23 multiple choice question test) improved significantly from an average score of 56 to 81% after the session (*p* < 0.01). Whilst our results are similar in finding an increase in knowledge immediately after the escape room, the authors did not test the students’ knowledge retention at a later date. Our results showed that whilst a third of students scored lower on their ‘2 weeks post-session test’ than immediately after the teaching, all students maintained a higher test score than before the teaching, indicating a level of knowledge improvement and retention.

**Table 4:** Healthcare themed escape rooms published in the literature with a method of outcomes and student demographics

Reference	Topic	Student level	Sample size and group size	Cost for materials	Designated time (min)	Outcomes assessed
Eukel H et al. 2017 [22]	Diabetes	Third year pharmacy students	*N* = 183; Teams of 5	NS	75	23 multiple choice question test 1 week prior to and immediately after the session. Additional survey regarding personal feedback on satisfaction and running of the escape room.
Backhouse A et al. 2019 [4]	Patient Safety	Third year medical students	*N* = 19; Teams of 6/7	£90.00	30	Post-session questionnaire regarding satisfaction and self-rated subjective increase in knowledge and confidence.
Gomez-Urquiza JL et al. 2019 [7]	Adult nursing	Second year nursing students	*N* = 105; Teams of 5	NS	30	Post-session questionnaire on satisfaction and self-rated knowledge improvement.
Guckian J et al. 2019 [12]	Dermatology	Third year medical students	*N* = 16; Teams of 4/5	NS	NS	Questionnaire issued before and after the escape room on subjective learning style preferences.
Kinio et al. 2019 [10]	Vascular surgery	First year medical students	*N* = 13; Teams of 3/4	NS	60	Questionnaire post-session on participant’s self-rated satisfaction, motivation, learning, communication and leadership skills. Further questionnaire 2 weeks later asking students about time spent reading pre-escape room supplementary material.
Jambhekar et al. 2019 [6]	Radiology	Radiology residents (various levels)	*N* = 144 Teams of 4–6	NS	60	Questionnaire post-session on participant’s self-rated satisfaction, motivation, learning relating to the event.
Cain J. 2019 [23]	Pharmacy	Third year pharmacology students	*N* = 141 Teams of 5/6	$12USD	45	Questionnaire post-session on students' perception of the escape room teaching method.
Current study	Paediatric radiology	Medical students (various levels)	*N* = 19 Teams of 6/7	£93.83	30	Single best answer test before, after and at two weeks post-teaching session + feedback questionnaire on the experience.

*NS* = not stated

With respect to student feedback, our findings are similar to other studies, reporting that the majority of participants enjoyed the experience of the escape room, and preferred this teaching to a didactic lecture [4, 7, 12, 22]. In studies by Backhouse et al. [4] and Gomez-Urquiza et al. [7], undergraduate medical and nursing students also self-reported feeling more confident in implementing new skills and motivated to learning more about the topic outlined by the escape room. Kinio et al. [10] also found that as a result of conducting their vascular surgery themed escape room, 92% of students reported an increased interest in the specialty.

Although difficult to quantify objectively, we feel that our teaching method was useful in providing ‘non-technical’ skills to participants that would otherwise not be incorporated in a traditional lecture-based tutorial, such as lateral thinking, making decisions under time pressure, effective communication and teamwork [24]. In future iterations of our escape room, to improve educational value, there are added aspects we would include. Taking the student feedback into account, these could comprise of extra puzzles to create added time pressures on the participants, more complex games (such as linking more than just two posters/objects in the room, or perhaps having only one person able to see and describe findings on radiographs to other team members, who then have to determine the diagnosis), and running sessions for undergraduates of a similar seniority level to encourage everyone’s active participation.

As with all studies, ours had some limitations. The first is due to the lack of student randomisation to either an escape room, lecture-based teaching or a ‘no directed teaching’ group, and the fact that our teaching session included a lecture component as well. Therefore, whilst we were able to demonstrate students’ enjoyment and improvement in paediatric radiology knowledge, we cannot draw any conclusion as to whether this teaching method is more effective than the usual lecture-based teaching or self-directed learning, nor the degree of influence the tutorial component of the escape room session played on knowledge improvement. Nevertheless, our student feedback suggested that the majority preferred an escape room with tutorial rather than a longer lecture and, given the importance of feedback in educational activities [24], we felt it would be crucial to include a ‘de-briefing’ tutorial to consolidate and highlight the learning objectives, rather than to assume the solving of the escape room conferred an understanding of radiology. Whether placing the tutorial before the escape room would have been more effective remains to be explored.

Secondly, we recruited students from different levels of training, with different prior levels of radiological knowledge and from different universities. Although the teaching was targeted at undergraduate students, we also had one recently qualified junior doctor. This meant some participants found the experience harder than others and perhaps did not feel they could participate in all the puzzles to the same degree. Nevertheless, even if unable to understand the puzzles, the escape room set-up promoted communication between the more junior participants with their seniors and allowed the supervising radiologist an opportunity to overhear discussions and questions students were asking each other. This enabled a more tailored approach to the post-escape room tutorial, as knowledge gaps could be better addressed, without the student having to explicitly announce them to the teacher, which can be intimidating. Despite the different student seniority levels in this study, all teams were able to solve the puzzles within a reasonable time frame, suggesting the teaching method does still work with mixed student abilities, although a more uniform ability level could improve satisfaction and participation.

Thirdly, we acknowledge that we had a small sample size, a short 8-question SBA test to assess knowledge and that not all students completed the 2 weeks post-session test. Whilst a larger student group, with those from a greater variety of medical schools, and more comprehensive testing would be ideal, given the experimental nature of this novel teaching method, we believe sufficient early positive results have been demonstrated to allow for more rigorous assessment in the future, should research in this aspect of medical education be pursued.

The puzzles used in our escape room have been provided in this article to allow others to replicate or modify this teaching method as wished. Since radiology lends itself well to problem-based puzzles, devising new games on other modalities, and on other subspecialty or general radiology topics could help improve undergraduate students’ perception and interest in our subspecialty [25, 26]. Further work should focus on comparing this teaching method with conventional lecture-based teaching to demonstrate its effectiveness. One should bear in mind that the use of this teaching method alone for radiology undergraduate teaching may pose some limitations given that only a limited aspect of the total curricula is outlined, and there may be practical issues in ‘scaling up’ the teaching to allow for larger student numbers. This could be partly overcome with technological innovations, such as converting several puzzles into an online web-based game, or a smartphone application to allow more students to play and learn at their own pace although it would come with its own limitations by reducing the interpersonal communication and interaction aspect of the escape room method. Nevertheless, we believe the ‘fun factor’ associated with the escape room may provide some variety to traditional lecture-based methods and help to improve student engagement with general radiology education.

In conclusion, we have shown that a paediatric radiology themed escape room was a feasible, enjoyable and educational method for engaging undergraduate medical students in radiology education. Further work with larger student groups and comparison with traditional teaching methods are required to better understand the full educational value of this technique.

## Supplementary information

**Additional file 1: Figure A1** Radiology based learning objectives determined at the outset of the teaching, prior to the development of the escape room and associated tutorial. These objectives described were based on the RCR undergraduate education ^1^ and the ESR U-curriculum(Module U-II-10 paediatric radiology) [2]. **Figure A2**: The Single Best Answer (SBA) Test provided to all participants before, immediately after and at two weeks post escape room themed radiology teaching. The answers to the quiz are as follows: 1b, 2d, 3b, 4d, 5d, 6b, 7b, 8a. Question 6 was based on the systematic review by Kemp et al^3^, demonstrating highest association with rib fractures with suspected physical abuse, but insufficient evidence to quantify probability of association with corner metaphyseal fractures. **Figure A3**: Escape room rules and regulations. These instructions were read to students prior to entering the escape room and a copy of the rules were also left on the central table within the escape room itself as a reminder of good behaviour. **Figure A4**: Escape room backstory. This fictional story was read to the participants prior to entering the escape room in order to ‘set the mood’ and create and fun and engaging atmosphere. **Figure A5**: Teaching feedback form completed by students after the escape room themed teaching session to assess levels of enjoyment, difficulty and design of the escape room. **Figure A6**: A list of inventory and equipment purchased in order to develop and set up the escape room themed teaching. The location for sourcing the material and the costs incurred (including postage costs where bought online) are provided. **Figure A7**: Individualised free text comments provided by participants on the feedback forms after the teaching sessions.

## Data Availability

Data generated through this study are all presented either in the main manuscript or in the appendix.

## Abbreviations

ESR: European Society for Radiologists
RCR: Royal College of Radiologists
SBA: Single best answer
SGUL: St George’s, University of London
UCL: University College London
