# Supplementary material for: Feasibility of a paediatric radiology escape room for undergraduate education
Source: Insights Imaging. 2020 Mar 19;11:50. doi: 10.1186/s13244-020-00856-9 (PMC7082448; doi:10.1186/s13244-020-00856-9)
Supplement: Supplementary file 1 — Additional file 1: Figure A1 Radiology based learning objectives determined at the outset of the teaching, prior to the development of the escape room and associated tutorial. These objectives described were based on the RCR undergraduate education 1 and the ESR U-curriculum(Module U-II-10 paediatric radiology) [2]. Figure A2: The Single Best Answer (SBA) Test provided to all participants before, immediately after and at two weeks post escape room themed radiology teaching. The answers to the quiz are as follows: 1b, 2d, 3b, 4d, 5d, 6b, 7b, 8a. Question 6 was based on the systematic review by Kemp et al3, demonstrating highest association with rib fractures with suspected physical abuse, but insufficient evidence to quantify probability of association with corner metaphyseal fractures. Figure A3: Escape room rules and regulations. These instructions were read to students prior to entering the escape room and a copy of the rules were also left on the central table within the escape room itself as a reminder of good behaviour. Figure A4: Escape room backstory. This fictional story was read to the participants prior to entering the escape room in order to ‘set the mood’ and create and fun and engaging atmosphere. Figure A5: Teaching feedback form completed by students after the escape room themed teaching session to assess levels of enjoyment, difficulty and design of the escape room. Figure A6: A list of inventory and equipment purchased in order to develop and set up the escape room themed teaching. The location for sourcing the material and the costs incurred (including postage costs where bought online) are provided. Figure A7: Individualised free text comments provided by participants on the feedback forms after the teaching sessions. [file 13244_2020_856_MOESM1_ESM.docx]

**Appendix: Supplementary Electronic Material**

**Figure A1:** Radiology based learning objectives determined at the outset of the teaching, prior to the development of the escape room and associated tutorial. These objectives described were based on the RCR undergraduate education ^1^ and the ESR U-curriculum(Module U-II-10 paediatric radiology) ^2^.

**
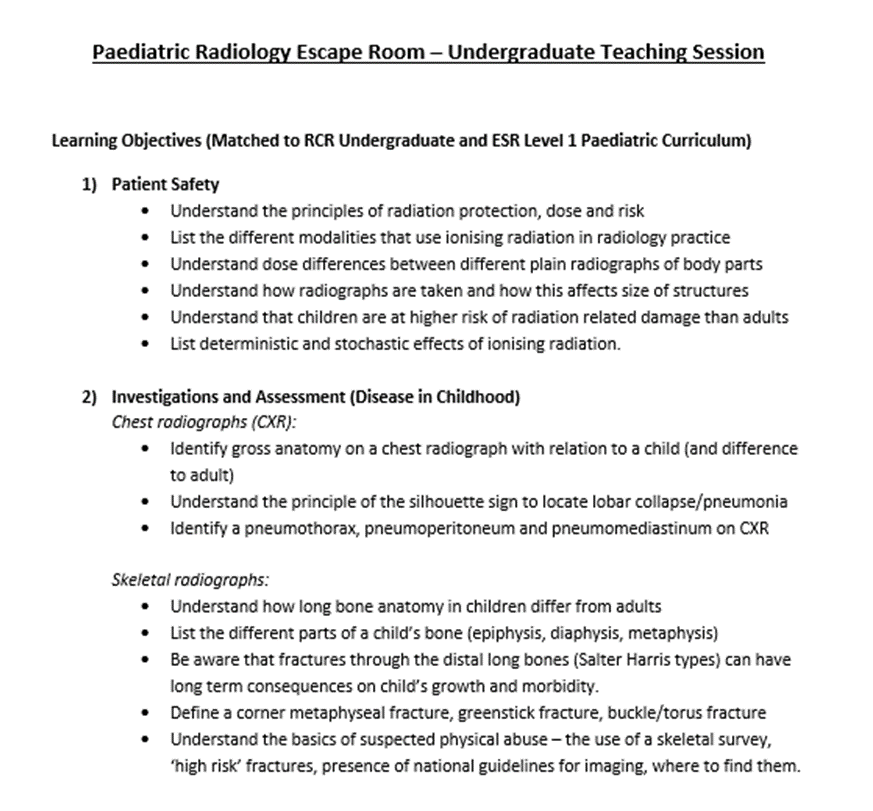
**

**Figure A2:** The Single Best Answer (SBA) Test provided to all participants before, immediately after and at two weeks post escape room themed radiology teaching. The answers to the quiz are as follows: 1b, 2d, 3b, 4d, 5d, 6b, 7b, 8a. Question 6 was based on the systematic review by Kemp et al^3^, demonstrating highest association with rib fractures with suspected physical abuse, but insufficient evidence to quantify probability of association with corner metaphyseal fractures.


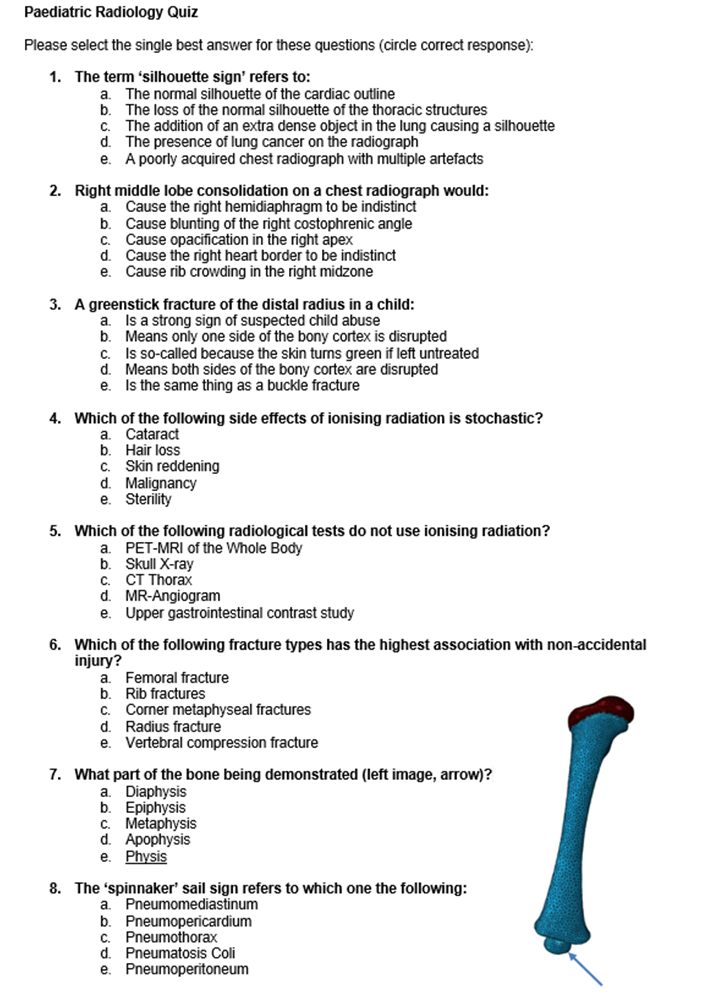


**Figure A3:** Escape room rules and regulations. These instructions were read to students prior to entering the escape room and a copy of the rules were also left on the central table within the escape room itself as a reminder of good behaviour.


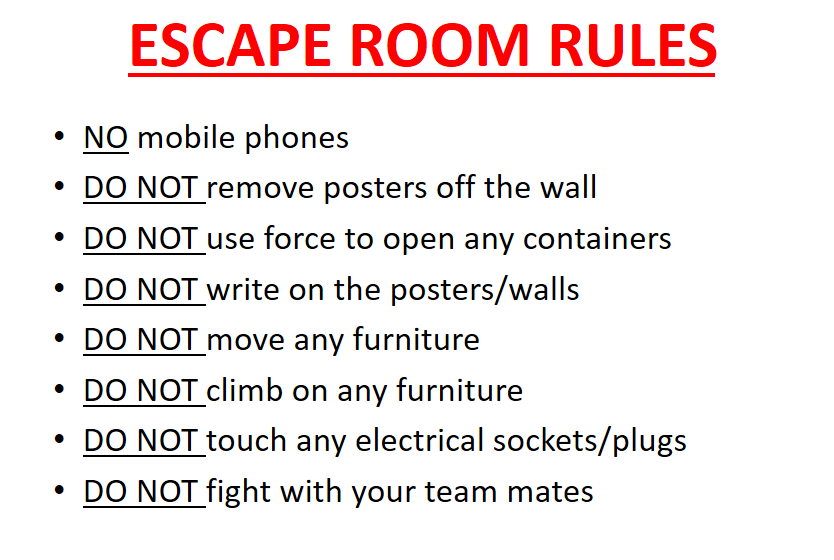


**Figure A4:** Escape room backstory. This fictional story was read to the participants prior to entering the escape room in order to ‘set the mood’ and create and fun and engaging atmosphere.

**
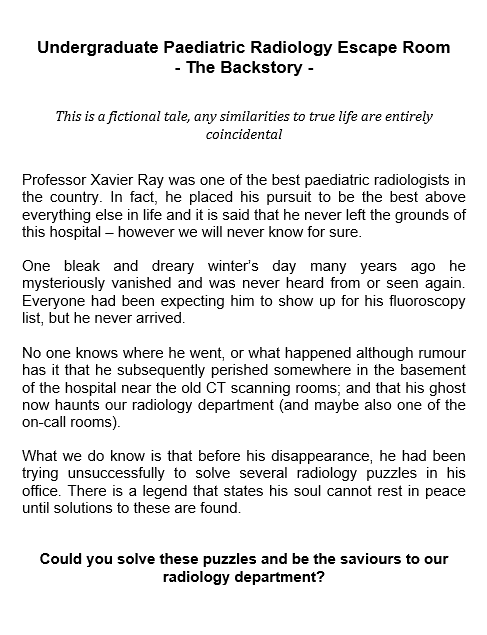
**

**Figure A5:** Teaching feedback form completed by students after the escape room themed teaching session to assess levels of enjoyment, difficulty and design of the escape room.

**
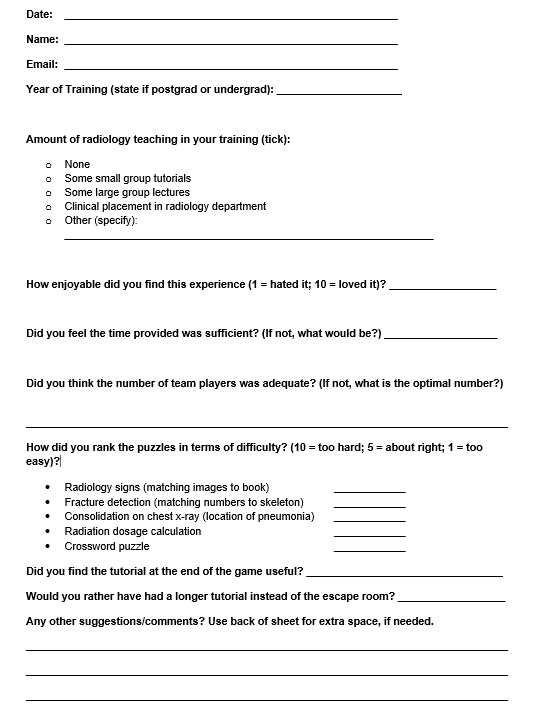
**

**Figure A6:** A list of inventory and equipment purchased in order to develop and set up the escape room themed teaching. The location for sourcing the material and the costs incurred (including postage costs where bought online) are provided.

**
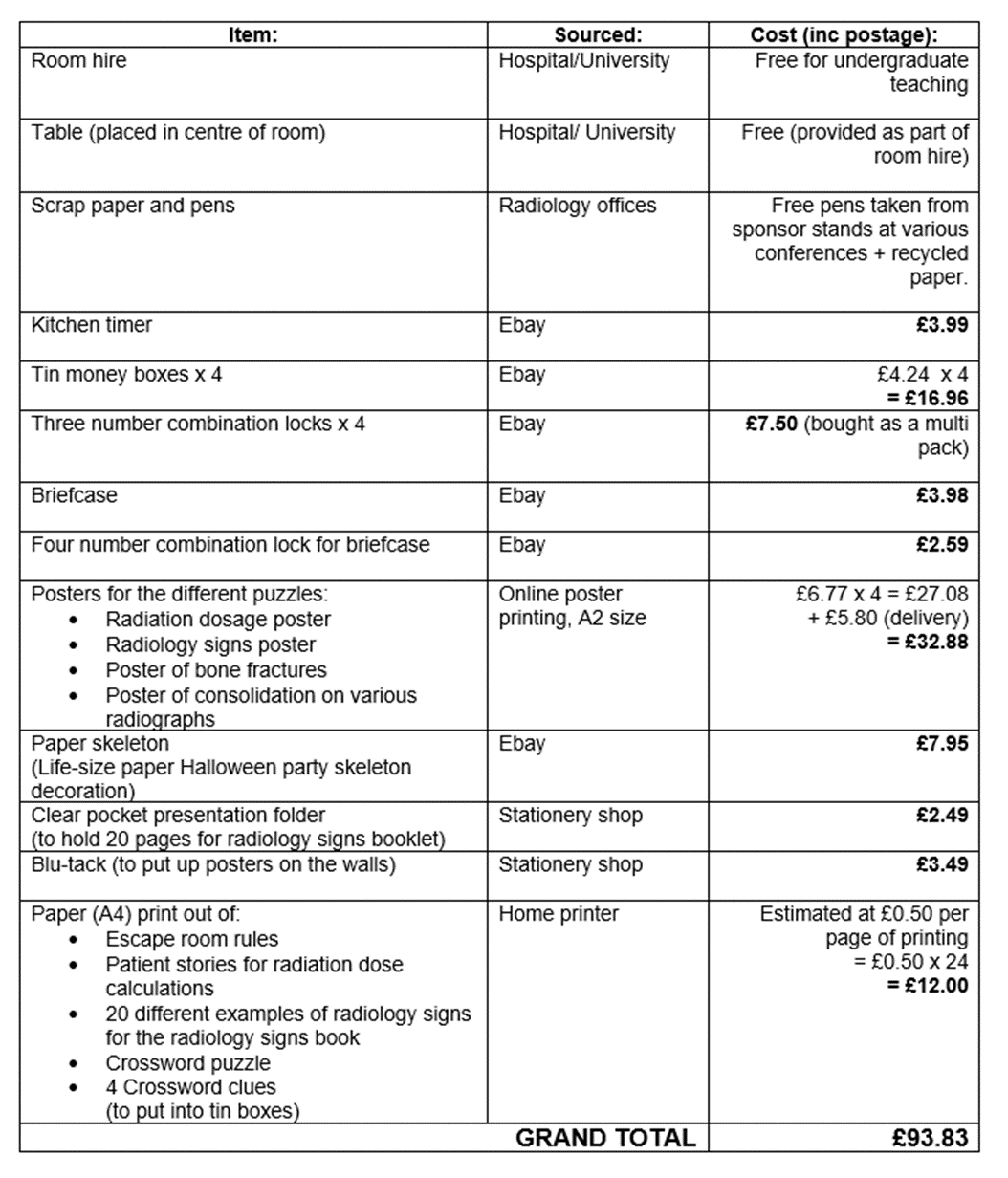
**

**Figure A7:** Individualised free text comments provided by participants on the feedback forms after the teaching sessions**.**

**Free Text Comments:**

- It was a really enjoyable teaching session and very engaging. Handout of slides would be useful but overall great
- I think a longer session would be useful as I felt that I learned actively in the escape room part and consolidated at the end. Explained basics better than I've experienced before!
- For the radiology sings matching exercise, it didn't require any actual knowledge - just matching pictures. May be more challenging to have the name of a sign and get us to match it to the x-ray
- Great imagination, exactly what we need to understand/remember radiology - can be monotonous otherwise.
- Thanks for this tutorial, I have learned a lot from this event
- Really good, no suggestions
- Thank you - very enjoyable and great way of learning
- Really enjoyed the structure of the session and the tutorial especially. Only slightly difficult aspect was balancing differences in knowledge between team players during escape room to ensure everyone was able to participate and understand what was going on
- More puzzles! Thanks for an enjoyable evening
- I think the escape room could be a bit harder but overall it was really good and really enjoyed it
- Maybe some additional elements to escape room but overall was brilliant
- Was really fun

**APPENDIX REFERENCES:**

1. The Royal College of Radiologists. Undergraduate radiology curriculum, second edition. 2017. https://www.rcr.ac.uk/sites/default/files/documents/undergraduate_radiology_curriculum_second_edition_2017.pdf (accessed 15th November 2019).

2. European Society of Radiology. Curriculum for Undergraduate Radiological Education. 2019. <https://www.myesr.org/sites/default/files/2019-02/ESR_2019TrainingCurriculum_Undergraduate_Edition%20March%202019.pdf> (accessed 15th November 2019).

3. Kemp AM, Dunstan F, Harrison S, et al. Patterns of skeletal fractures in child abuse: systematic review. *BMJ (Clinical research ed)* 2008; **337**: a1518.
